# Trajectories of Cognitive Symptoms in Sick-Listed Cancer Survivors

**DOI:** 10.3390/cancers13102444

**Published:** 2021-05-18

**Authors:** Kete M. Klaver, Sanne B. Schagen, Jacobien M. Kieffer, Allard J. van der Beek, Saskia F. A. Duijts

**Affiliations:** 1 Division of Psychosocial Research and Epidemiology, Netherlands Cancer Institute, 1066 CX Amsterdam, The Netherlands; s.schagen@nki.nl (S.B.S.); j.kieffer@nki.nl (J.M.K.); 2 Department of Public and Occupational Health, Amsterdam Public Health Research Institute, Amsterdam UMC, Vrije Universiteit Amsterdam, 1081 BT Amsterdam, The Netherlands; a.vanderbeek@amsterdamumc.nl; 3 Brain and Cognition Group, Psychology Research Institute, University of Amsterdam, 1018 WS Amsterdam, The Netherlands; 4 Netherlands Comprehensive Cancer Organisation (IKNL), 3511 DT Utrecht, The Netherlands

**Keywords:** cognitive symptoms, cancer, work ability, sick leave

## Abstract

**Simple Summary:**

The effects of cognitive symptoms on the ability to work are of major concern for cancer survivors. The aim of our study was to explore trajectories of cognitive functioning in sick-listed cancer survivors with work capacity. We found that cognitive functioning improved between two and four years after first day of sick leave, although cognitive symptoms remained of clinical concern in cancer survivors who are non-durable work-disabled (partly or fully). This underlines the importance to provide partly and fully, non-durable work disabled cancer survivors with evidence-based treatment options for their self-perceived cognitive symptoms.

**Abstract:**

Many non-central nervous system (CNS) cancer survivors experience cognitive symptoms, which may affect their self-perceived work ability. Little is known about trajectories of self-perceived cognitive functioning in cancer survivors in the period after work disability assessment. Therefore, we evaluated: (1) trajectories of self-reported cognitive functioning, in cancer survivors with work capacity, (2) differences in trajectories of self-reported cognitive functioning between three work disability groups, and (3) explanatory factors of trajectories of self-reported cognitive functioning. Participants (n = 206) were assessed on self-reported cognitive functioning at three time points between two and four years after first day of sick leave. A statistically significant improvement in cognitive functioning was found in the total group (β = 4.62, SE = 0.91, *p* < 0.001). When comparing cancer survivors in different work disability groups, similar trajectories of cognitive functioning were observed. Fatigue was the only factor found to be associated with the reported trajectory (β = −0.23, SE = 0.086, *p* = 0.08). Self-perceived cognitive functioning scores remained considerably lower than the mean score of the general Dutch population, indicating that cognitive symptoms are a persistent problem in sick-listed cancer survivors and that evidence-based treatment options are warranted.

## 1. Introduction

The past decades have seen rapid advances in cancer treatment, resulting in improved survival rates [1]. About 40–50% of cancer survivors are diagnosed at a working age [2]. Long term effects of cancer and cancer treatment on the ability to work are of major concern for cancer survivors and their family [3]. About 89% (range 84–94%) of cancer survivors, who were employed at the time of diagnosis, is able to (partly) return to work (RTW) within 2 years after diagnosis [4]. However, previous studies showed that cancer survivors have less working hours, higher unemployment rates, and higher work disability rates than their healthy counterparts [5]. Therefore, exploring and understanding factors associated with work disability within the growing community of cancer survivors is of utmost importance [1].

Previous (mainly cross-sectional) literature suggests that cognitive symptoms (i.e., problems with memory, concentration and executive functioning) are primary factors affecting self-perceived work ability in non-central nervous system (CNS) cancer survivors who have returned to work [6,7,8,9,10]. Previous literature also suggests that cognitive symptoms are associated with a higher risk of leaving the workforce [10,11,12]. Furthermore, qualitative research showed that about 41% of cancer survivors reported a reduced number of weekly working hours compared to pre-treatment levels because of cognitive symptoms [6]. These findings underline the importance of supporting cancer survivors with work capacity to cope with their cognitive symptoms. Such support could sustain their employability and prevent long-term work disability.

Worldwide, work disability assessment is used to estimate if a patient meets the requirements for receiving a work disability benefit. In the Netherlands, level of work disability of sick-listed employees is assessed by an insurance physician (IP) of the Dutch Social Security Agency (SSA), two years after the first day of sick leave. Based on the IP’s report on work (in)capacity of the employee, a labour expert calculates the loss of former wages earned, by comparing former wages earned to potential wages of possible jobs according to current work capacity. Sick-listed employees are granted a work disability benefit if loss of former wages exceeds 35% [13]. According to the Dutch law, work disability is divided into four groups: (1) able to work (i.e., less than 35% loss of former wages earned), (2) partly, non-durable work disabled (i.e., between 35 to 80% loss of former wages earned), (3) fully, non-durable work disabled (i.e., between 80 to 100% loss of former wages earned), and (4) fully, durable work disabled (i.e., between 80 to 100% loss of former wages earned; no change expected) [14]. Those in the first group (i.e., able to work) do not receive work disability benefits; moreover, those in the second and third group are considered to be (partly) able to RTW, and earn a salary and receive a (partly) work disability benefit [14]. Although patients in those three groups are expected to (partly) RTW, the severity of cognitive symptoms in the period after work disability assessment is unclear. Due to the impact of cognitive symptoms on work ability, severity of cognitive symptoms may differ between the work disability groups. 

To date, only few longitudinal studies have investigated the association between cognitive symptoms and work ability in cancer survivors [6,15,16]. Dorland et al. (2018) found that self-perceived cognitive symptoms in employed cancer survivors persisted and were consistently related to work limitations over the first 18 months after returning to work [15]. However, little is known about the trajectory of cognitive symptoms in sick-listed cancer survivors with work capacity, in the period following work disability assessment (i.e., from 24 months follow-up onward), while this information is crucial to identify patients who may benefit from supportive care options in order to sustain their work ability. Further, previous studies have shown that fatigue and depressive symptoms are associated with cognitive symptoms [11,17,18,19], and that these symptoms are also negatively associated with work functioning over time [20]. Also, treatment-related characteristics (e.g., treatment modality, intensity, time since completion of treatment), sociodemographic characteristics (e.g., educational level), and coping style have been reported to potentially influence cognitive symptoms as well [21,22,23,24]. Hence, the trajectory of cognitive symptoms in sick-listed cancer survivors with work capacity should be investigated taking into consideration its association with these factors.

In this study, we used data from a prospective cohort of Dutch cancer survivors approaching two years of sick leave. Cancer survivors were monitored up to four years after their first day of sick leave [13]. The objectives of the current study were to explore in cancer survivors with work capacity: (1) trajectories of self-reported cognitive functioning, between two and four years after first day of sick leave, in cancer survivors with work capacity, (2) differences in trajectories of self-reported cognitive functioning between the three work disability groups of cancer survivors with work capacity, and (3) explanatory factors of trajectories of self-reported cognitive functioning. Results of this study may help to identify sick-listed cancer survivors who may benefit from cognitive rehabilitation interventions to cope with cognitive symptoms, and therefore sustain and/or improve their work ability.

## 2. Materials and Methods

### 2.1. Study Sample and Procedure

We used data from a prospective cohort study. The aim of this prospective cohort study was to identify prognostic factors associated with work disability in sick-listed cancer survivors. Patients who approached the maximum term of 2 years sick leave and applied for a work disability benefit were selected from the SSA registries, and were followed for two years (i.e., up to four years after first day of sick leave). A detailed description of the initial prospective cohort study has been reported elsewhere [13,25].

For the current study, we included cancer survivors who: (1) had a confirmed diagnosis of non-CNS cancer at baseline, (2) had an employment contract at time of diagnosis, (3) were occupationally active (i.e., had a paid employment contract or were involved in therapeutic work (i.e., work that allows for a gradual and flexible build-up of workload and working hours)), and (4) were between 18 and 64 years of age when approaching two years of sick leave. Also, for the current study, a selection was made of cancer survivors with work capacity, based on their work disability assessment (i.e., cancer survivors who are: (1) able to work (i.e., less than 35% loss of former wages earned), (2) partly, non-durable work disabled (i.e., between 35 to 80% loss of former wages earned), or (3) fully, non-durable work disabled (i.e., between 80 to 100% loss of former wages earned)). Hence, those assessed as (4) fully, durable work disabled (i.e., between 80 to 100% loss of former wages earned; no change expected) were excluded. Cancer survivors were also excluded if they were still receiving chemotherapy and/or radiotherapy. Furthermore, in order to sustain homogeneity regarding work situation (e.g., employer security, return to work situation, vulnerability for work disability), cancer survivors who were self-employed, were working in a sheltered workplace (i.e., a workplace that employs workers with disabilities), or were registered as ‘social security safety netters’ (i.e., workers whose temporary employment contract ended during sick leave, temporary agency workers and workers who are unemployed [25]) were excluded.

At baseline (i.e., at two years after first day of sick leave (T0)), potentially eligible participants received a package, by post at their home address, that included an information leaflet, a baseline questionnaire including questions on sociodemographic characteristics, treatment-related characteristics, and patient-reported outcomes, as well as an informed consent form. Follow-up questionnaires on patient-reported outcomes were sent one year (T1), and two years (T2) after baseline.

### 2.2. Measurements

#### 2.2.1. Sociodemographic Characteristics

Sociodemographic characteristics, including age, gender and education, were obtained via questionnaire. A questionnaire was used to acquire information on general employment issues and on work accommodation, including employment sector, type of employment (fixed/temporary), years of work experience, and working hours and days per week according to employment contract.

#### 2.2.2. Clinical and Treatment-Related Characteristics

Clinical information, including cancer site, month/year of diagnosis, received (and future) treatment(s), (i.e., surgery, radiation, chemotherapy, immunotherapy, targeted treatment, hormonal therapy, other medication), and recurrence(s) of the disease, were obtained via self-report.

#### 2.2.3. Work Disability

The level of work disability, expressed as the first three categories based on wage loss in percentage of former wage, was retrieved from the SSA: (1) able to work (i.e., less than 35% loss of former wages earned), (2) partly, non-durable work disabled (i.e., between 35 to 80% loss of former wages earned), and (3) fully, non-durable work disabled (i.e., between 80 to 100% loss of former wages earned). The classification of work disability did not change over time.

#### 2.2.4. Patient-Reported Outcomes

##### Cognitive Functioning

Self-perceived cognitive functioning was measured using the Cognitive Functioning scale of the Dutch validated version of the EORTC QLQ C-30 [26]. This scale consists of two items assessing difficulty concentrating and memory problems. Items are scored on a 4-point scale ranging from 1 (not at all), to 4 (very much). The scores were reversed and linearly transformed to a scale ranging from 0 to 100. A higher score indicates a better cognitive functioning.

##### Fatigue, Depression and Coping

Fatigue was measured using the Functional Assessment of Chronic Illness therapy-Fatigue (FACIT-F) [27]. This questionnaire consists of 13 items. Items are scored on a 5-point scale, ranging from not at all to very much. A higher score indicates less fatigue (range 0–52).

Depression was measured using the 20 item Center for Epidemiologic Studies depression Scale (CES-D) [28]. Items are scored on a 4-points scale, ranging from none of the time to most of the time. A higher score indicates more depressive symptoms (range 0–60).

Coping was measured using the 47-item Utrecht Coping List (UCL) [29]. In total, the UCL consists of 7 subscales that measure different coping strategies: active tackling, seeking social support, palliative reacting, avoiding, passive reacting, reassuring thoughts and expression of emotions [30]. Items are scored on a 4-point scale, ranging from ‘hardly ever’ to ‘very often’. A higher score on a subscale indicates an increased tendency using that specific coping strategy. Scoring ranges differ per coping strategy.

### 2.3. Statistical Analysis

Descriptive statistics were used to characterize the study sample. Scores on the patient-reported outcomes questionnaires were calculated according to published scoring algorithms. To evaluate the trajectory of cognitive functioning, we used a mixed modelling approach based on maximum likelihood estimation with random intercept and slope. This approach takes into account the within and between person variability, and deals adequately with missing data [31]. Little’s Test was used to check the assumption for missing data completely at random [32]. We checked for the presence of a linear effect of time and added a quadratic term to determine whether an initial improvement or deterioration in cognitive functioning was followed by a deceleration of this effect. The choice between covariance structures (i.e., unstructured, compound symmetry, and autoregressive) and a model including only a linear effect of time and a model including both a linear and quadratic effect of time was based on model fit statistics: the Bayesian information criterion (BIC) and Akaike information criterion (AIC) [33,34].

Normative data for the Cognitive Functioning scale of the EORTC QLQ C-30 was used to interpret cognitive functioning scores, whereby the mean cognitive functioning score of the general Dutch population is 93 [35]. We also used a threshold for clinical importance, based on external criteria reflecting the clinical relevance of the problem (i.e., a score below 75 indicates clinically important cognitive symptoms) [36].

We determined whether trajectories of cognitive functioning scores differed between cancer survivors in the three included groups of work disability. Differences in change from baseline to follow-up between groups were accompanied by effect sizes (ES). ES were calculated based on the estimated marginal means. Effect sizes of 0.2 were considered small, 0.5 moderate, and 0.8 large. Effect sizes of ≥0.5 were considered to be clinically relevant [37].

Finally, we performed exploratory analysis in which we evaluated the following potential explanatory factors of cognitive functioning trajectories: (1) sociodemographic characteristics (i.e., educational level), (2) treatment-related characteristics (i.e., treatment modality, intensity and time since completion of treatment), and (3) patient-reported outcomes (i.e., fatigue, depression and coping style). All effects were considered statistically significant at a *p*-value smaller than 0.05. Statistical analyses were performed using Statistical Package for the Social Sciences (SPSS), version 26 (SPSS Inc., IBM Corp., Armonk, NY, USA).

## 3. Results

### 3.1. Sample Characteristics

Recruitment and participant flow of the original cohort have been reported in detail elsewhere [25]. Based on inclusion criteria for the current study, a total of 206 cancer survivors (62 men and 144 women) with a mean age of 51.0 years (SD 6.8; range 28–63 years) were included in the analyses. Information on baseline sociodemographic and treatment-related characteristics of the sample are provided in Table 1. Breast cancer was the most prevalent cancer type among female cancer survivors (n = 104; 72%). Urogenital cancer was the most prevalent cancer type among male cancer survivors (n = 10; 16%). The mean time since diagnosis at baseline was 23.2 months (SD 9.3; range 15–98 months). Cancer survivors worked on average up to 31.9 h/week (S.D 7.9, range 7–48 h), and 68% of the cancer survivors (n = 141) worked full time at time of cancer diagnosis. Loss to follow-up at T1 and T2 were 13% (n = 26) and 16% (n = 33), respectively.

Mean and standard deviations of cognitive functioning for the total group and for the three work disability groups separately can be found in Table 2.

### 3.2. Trajectory of Cognitive Functioning

**Figure 1:**
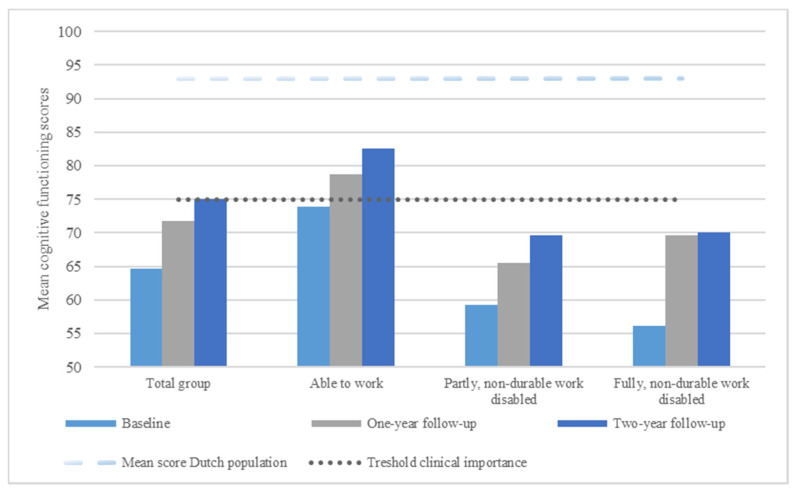
Mean cognitive functioning scores for the total group and separate work disability group.

After inspection of the fit indices (AIC and BIC), we found that the model including a linear effect of time showed the best fit for the total group. A statistically significant improvement in cognitive functioning was observed (β = 4.62, SE = 0.91, *p* < 0.001). The effect size for change in cognitive functioning in the total group was 0.26 at one-year follow-up (T0-T1), and 0.36 at two-year follow up (T0-T2). The negative correlation between the intercept and the linear growth parameter (β = −100.38, SE = 50.05, *p* = 0.045) indicated that cancer survivors with relatively high cognitive functioning at baseline had a slower linear increase, whereas cancer survivors with low cognitive functioning at baseline had a faster linear increase in cognitive functioning. Cognitive functioning scores of the total group (Figure 1) were below the mean cognitive functioning score of the general Dutch population at all timepoints. Furthermore, cognitive functioning scores of the total group were below the threshold of clinical importance at T0 and T1, and equal to this threshold at T2. 

### 3.3. Level of Work Disability and Trajectories of Cognitive Functioning

**Figure 2:**
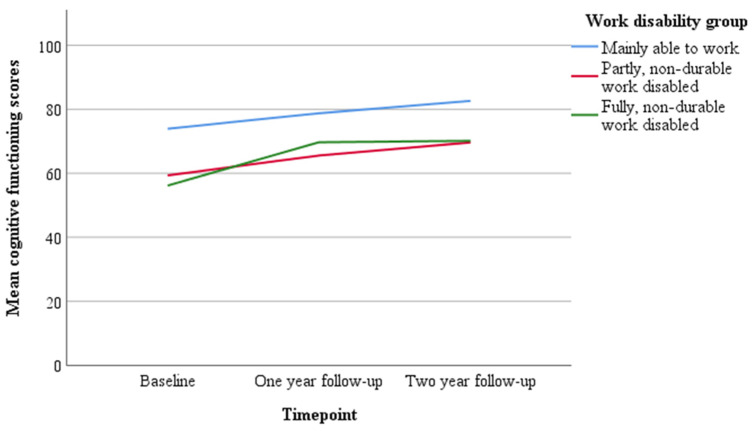
Trajectories of cognitive functioning for separate work disability group.

No significant difference in changes in cognitive functioning between the three groups were found (Figure 2). At baseline, however, cancer survivors who are non-durable work disabled (either party or fully) had significantly worse cognitive functioning compared to cancer survivors who are considered able to work (i.e., those below the 35% loss of former wages threshold) (β = −14.26, SE = 5.30, *p* = 0.008 and β = −19.74, SE = 6.75, *p* = 0.004, respectively). Cognitive functioning scores of the separate work disability groups (Figure 1) were below the mean cognitive functioning score of the general Dutch population at all timepoints. Furthermore, cognitive functioning scores of cancer survivors who are considered able to work, were below the threshold of clinical importance at T0 and above this threshold at T1 and T2. Cognitive functioning scores of cancer survivors who are non-durable work disabled (either party or fully), were below the threshold of clinical importance at all timepoints.

### 3.4. Factors Associated with Trajectories of Cognitive Symptoms

In the total group, educational level was not a significant predictor of changes in cognitive functioning. Neither were treatment-related characteristics, such as treatment modality, treatment intensity (e.g., local versus systemic treatment) and time since completion of treatment (Table 3). In addition, educational level and treatment-related characteristic were not associated with cognitive functioning at baseline. More fatigue at baseline was associated with lower cognitive functioning at baseline (β = 1.53, SE = 0.21, *p* < 0.001). Furthermore, changes over time in cognitive functioning were predicted by baseline fatigue; more fatigue at baseline was associated with a faster improvement of cognitive functioning (β = −0.23, SE = 0.086, *p* = 0.008). Depressive symptoms at baseline were associated with worse cognitive functioning at baseline (β = −1.53, SE = 0.22, *p* < 0.001), but were not a significant predictor of the changes over time in cognitive functioning. Higher scores on the coping subscales passive reaction and expression of emotion at baseline were associated with lower cognitive functioning at baseline (β = −2.43, SE = 0.71, *p* = 0.001 and β = −4.93, SE = 1.64, *p* = 0.003, respectively). However, subscales of coping did not significantly predict changes over time in cognitive functioning.

## 4. Discussion

This is the first study exploring trajectories of self-perceived cognitive functioning in sick-listed cancer survivors with work capacity. In the total group, cognitive functioning improved after work disability assessment (i.e., between two and four years after first day of sick leave), although the effect size of the change in cognitive functioning over time was small to medium. When comparing cancer survivors in the three groups of work disability, quite similar trajectories of cognitive functioning were observed. Fatigue at two years after first day of sick leave was the only factor found to be associated with the reported trajectory of cognitive functioning.

In our study, we found a small to medium change in cognitive functioning over time in a group a sick-listed cancer survivors with work capacity. Mean baseline and follow-up cognitive functioning scores of our sample are of clinical concern, according to the threshold for clinical importance established by Giesinger et al. [36]. When compared to the mean cognitive functioning score of the general Dutch population [35], their scores are considerably lower. Together, these observations indicate that cognitive symptoms are a serious and long-term problem in sick-listed cancer survivors with assessed work capacity.

Cancer survivors who were non-durable work disabled (party or fully) reported worse cognitive functioning compared to those assessed as being able to work, two years after first day of sick leave. Cognitive functioning improved to a clinically acceptable level in this latter group, whereas cognitive functioning continued to be of clinical concern for the non-durable work disabled cancer survivors. In practice, this means that in those survivors who are able to work, recovery takes place up to four years after the first day of sick leave, whereas in non-durable work disabled (partly or fully) survivors, expectations for recovery are lower based on our results.

A number of factors were found to be associated with cognitive functioning at two years of sick leave; cancer survivors with higher levels of fatigue, depression, passive reactive coping, and emotionally expressive coping, reported worse cognitive functioning. These findings are consistent with previous research demonstrating fatigue, mood and coping to be associated with cognitive symptoms in cancer patients [15,23,24,38], suggesting that these symptoms are likely to co-occur and cluster [39]. Fatigue was the only factor found to be (weakly) associated with subsequent change in cognitive functioning. Potentially, patients with more fatigue at baseline may have had more room for improvement, whereas patients with less fatigue may improve to a smaller degree. Since fatigue is associated with cognitive symptoms, patients with more fatigue at baseline might exhibited a stronger decrease of cognitive symptoms over time. This finding is in line with several (intervention) studies, in which it was demonstrated that lower baseline symptom levels are predictive of improvement of symptoms over time [40,41,42,43,44]. However, the association found in current study needs to be interpreted with caution due to the small effect.

In previous literature, it has been demonstrated that cognitive symptoms may occur in patients receiving systemic cancer treatments, such as chemo-, endocrine- and immunotherapy. Local treatment (i.e., surgery and/or radiotherapy) may also be associated with cognitive symptoms, however this association has been found to be less pronounced [18,45,46]. The current study found no association between treatment-related characteristics (i.e., treatment modality, intensity, and time since completion of treatment) and trajectories of self-perceived cognitive functioning. There are several possible explanations for this result. At first, the majority (84%) of our sample received systemic cancer treatment and it is therefore difficult to investigate a potential differential impact of therapies on changes in cognitive function. Furthermore, cancer survivors without cognitive symptoms may have already returned to work before two years of sick leave, and thus did not need to apply for a work disability grant. In that case, our sample might reflect cancer survivors with a more unfavorable prognosis as to cognitive functioning, regardless of cancer treatment-related characteristics. Another possible explanation for this finding may be that functional status (e.g., physical, emotional, cognitive, social functioning) of cancer patients prior to cancer treatment may predict the trajectory of self-perceived cognitive symptoms over time, rather than cancer treatment-related characteristics [47]. This indicates the importance of assessing functional status and symptom burden prior to treatment if aiming at identifying patients vulnerable for cognitive symptoms.

A strength of this study is its longitudinal design, which made it possible to identify trajectories of cognitive symptoms with follow-up up to four years after the first day of sick leave of these cancer survivors. Furthermore, as a result of identification of participants from the SSA registries, the current study included cancer survivors, assessed for work disability after two years of sick leave, from the entire Dutch working population [14].

Our study had several limitations. First, the division of the cohort into three groups in terms of level of work disability resulted in relatively small fully, non-durable work disabled group of cancer survivors (n = 38), and may have underestimated the effect of level of work disability on the trajectory of cognitive symptoms. A second limitation was the absence of neuropsychological assessment to measure cognitive functioning. However, cognitive functioning assessed using self-reports may be more sensitive to fluctuations in fatigue and mood than neuropsychological assessment [19].

The results of current study underscore the importance to provide partly and fully, non-durable work disabled cancer survivors with evidence-based treatment options for their self-perceived cognitive symptoms, especially since this group of cancer survivors is expected to (partly) RTW based on the assessment of the SSA. So far, several intervention studies for cognitive symptoms in cancer survivors have been conducted [19,48]. However, due to methodological limitations (e.g., small sample sizes, limited control groups, lack of inclusion criteria regarding cognitive functioning) and absence of work-related outcome measures, no firm conclusions can be drawn from these intervention studies and further research into the effectiveness of interventions for cognitive symptoms in cancer survivors with work capacity is needed. Furthermore, our findings suggest that if improvement of cognitive symptoms is a desired outcome, management of cognitive symptoms together with fatigue, mood and coping may be a beneficial approach. Currently, an RCT is underway that evaluates such an approach for cognitive symptoms at work [49].

## 5. Conclusions

Our results indicated that cognitive symptoms are of clinical concern in sick-listed cancer survivors with work capacity, up to four years after the first day of sick leave. Although the cognitive symptoms improved over time, cognitive functioning continued to be of clinical concern in cancer survivors who are non-durable work-disabled (partly or fully). Therefore, this population is in need of options to manage their cognitive symptoms and thereby improve their ability to RTW and sustain their employability at the longer term.

## Figures and Tables

**Table 1:** Sociodemographic, clinical and treatment-related characteristics at baseline.

	Total
	N = 206
Sociodemographic characteristics	
Age at T0 (years): mean (SD)/range	51.0 (6.8)/28–63
Gender, N (%)	
Male	62 (30)
Female	144 (70)
Marital status, N (%)	
Single	22 (11)
Married	135 (66)
Living with partner	27 (13)
Divorced/widowed	22 (11)
Education, N (%)	
None/primary/lower vocational	47 (20)
Secondary school	29 (14)
Vocational education/upper secondary school	67 (33)
Upper vocational education/university	63 (31)
Clinical and treatment-related characteristics at baseline	
Cancer type, N (%)	
Breast	104 (51)
Digestive—colon	11 (5)
Digestive—other	13 (6)
Head and Neck	12 (6)
Hodgkin lymphoma	3 (2)
Non-Hodgkin lymphoma	11 (5)
Hematologic	11 (5)
Respiratory	9 (4)
Urogenital (female/male)	20 (10)
Urinary tract	8 (4)
Endocrine	2 (1)
Dermatologic	1 (0.5)
Locomotor (bone/sarcoma)	1 (0.5)
Metastasis, N (%)	
Lymph node	78 (38)
Distance	21 (10)
None	104 (51)
Time since diagnosis (months): mean (SD)/range	23.2 (9.4)/15–98
Being free of disease, N (%)	
Yes	108 (52)
Don’t know	56 (27)
No	39 (19)
Treatment, N (%)	
Surgery	154 (75)
Chemotherapy	148 (72)
Hormonal therapy	77 (37)
Immunotherapy	20 (10)
Radiotherapy	124 (60)
None	2 (1)
Time since completion treatment (months): mean (SD)/range	13.7 (6.5) 1–49
Ongoing treatment, N (%)	78 (38)
Surgery	6 (3)
Hormonal therapy	67 (33)
Immunotherapy	4 (2)
Stem cell transplant	1 (0.5)
Treatment intensity, N (%)	
Local treatment only (surgery and/or radiotherapy)	30 (16)
Systemic treatment (whether or not part of combination therapy)	173 (84)
Work-related characteristics—at time of diagnosis	
Sector, N (%)	
Education	21 (10)
Business and financial	21 (10)
Industry	28 (14)
Health care	69 (34)
Trade	17 (8)
Public services	12 (6)
Transport	12 (6)
Other	26 (13)
Working hours per week: mean (SD)/range	31.9 (7.9)/7–48
Full time, N (%)	141 (68)
Shift work, N (%)	
Yes	68 (33)
No	137 (67)
Managerial tasks, N (%)	
Yes	45 (22)
No	160 (78)
Job tenure, N (%)	
≤10 years	101 (49)
>10 years	105 (51)
Work-related characteristics—at baseline	
Work disability, N (%)	
<35%	84 (41)
35–80%	84 (41)
>80%	38 (18)

**Table 2:** Means and standard deviations of cognitive functioning for the total group and separate work disability groups.

	Total Group		Able to Work		Partly, Non-Durable Work Disabled		Fully, Non-Durable Work Disabled	
	N	Mean (SD) *	N	Mean (SD) *	N	Mean (SD) *	N	Mean (SD) *
Baseline	206	64.6 (27.2)	84	73.9 (22.7)	84	59.3 (26.0)	38	56.1 (32.7)
One-year follow-up	180	71.7 (26.1)	73	78.7 (23.4)	74	65.5 (25.3)	33	69.7 (30.2)
Two-year follow-up	173	75.0 (23.8)	71	82.6 (22.1)	73	69.6 (22.3)	29	70.1 (27.2)

* means and standard deviations of cognitive functioning.

**Table 3:** Explanatory factors of the trajectory of cognitive functioning of the total group.

						CI	
Factors	Beta	SE	Df	T	*p*	Lower Bound	Upper Bound
Sociodemographic characteristics							
Educational level *							
Medium	2.36	5.86	198.23	0.40	0.69	−9.19	13.92
High	1.97	5.80	198.96	0.34	0.74	−9.47	13.40
Treatment-related characteristics							
Treatment modality (yes/no) **							
Surgery	1.52	2.13	180.52	0.71	0.48	−2.69	5.73
Chemotherapy	2.47	2.00	175.48	1.24	0.22	−1.48	6.43
Hormonal therapy	1.32	1.87	176.54	0.70	0.48	−2.38	5.01
Immunotherapy	0.38	3.16	186.01	0.12	0.91	−5.86	6.61
Radiotherapy	1.32	1.86	177.04	0.71	0.48	−2.35	4.98
Treatment intensity							
Local treatment only (surgery and/or radiotherapy)	−0.94	2.53	172.22	−0.37	0.71	−5.94	4.07
Systemic treatment (whether or not part of combination therapy)	0.44	2.43	171.78	0.18	0.86	−4.37	5.24
Time since completion treatment	0.28	0.18	83.99	1.50	0.14	−0.090	0.65
Patient-reported outcomes							
Fatigue	−0.23	0.086	178.42	−2.70	0.008	−0.40	−0.062
Depression	0.18	0.093	186.12	1.89	0.061	−0.0082	0.36
Coping							
active tackling	−0.26	0.24	179.17	−1.048	0.30	−0.74	0.23
seeking social support	0.28	0.28	173.04	1.021	0.31	−0.26	0.83
palliative reacting	0.071	0.26	174.57	0.28	0.78	−0.44	0.58
avoiding	−0.028	0.30	178.19	−0.095	0.92	−0.62	0.56
passive reacting	0.24	0.28	186.22	0.87	0.39	−0.31	0.79
reassuring thoughts	0.49	0.33	178.64	1.50	0.14	−0.16	1.14
expression of emotions	0.89	0.62	181.75	1.44	0.15	−0.34	2.12

* Low educational level was used as the reference group. ** No treatment (with the treatment modality of interest) was used as the reference group.

## Data Availability

The dataset used and analyzed during the current study will be available from the corresponding author (stored in a data repository at the Amsterdam University Medical center) on reasonable request.

## Abbreviations

The following abbreviations are used in the manuscript:

AIC	Akaike information criterion
BIC	Bayesian information criterion
CES-D	Center for Epidemiologic Studies Depression Scale
CNS	Central nervous system
EORTC QLQ	European Organization for Research and Treatment of Cancer—Quality of Life questionnaire
ES	Effect Size
FACIT-F	Functional Assessment of Chronic Illness Therapy—Fatigue
IP	Insurance phycisian
RTW	Return to work
SD	Standard Deviation
SSA	Social Security
UCL	Utrecht Coping List
